# Effect of SGLT-2 inhibitor, dapagliflozin, on left ventricular remodeling in patients with type 2 diabetes and HFrEF

**DOI:** 10.1186/s12872-023-03591-3

**Published:** 2023-11-08

**Authors:** Qianyu Fu, Longhua Zhou, Yuqin Fan, Fenfen Liu, Yuanyuan Fan, Xin Zhang, Li Wang, Lan Cheng

**Affiliations:** Jiangxi Provincial People’s Hospital, The First Affiliated Hospital of Nanchang Medical College, Nanchang, Jiangxi China

**Keywords:** Dapagliflozin, Type 2 Diabetes, Heart Failure with reduced ejection fraction, Left ventricular remodeling, Echocardiography

## Abstract

The current study evaluated the effect of SGLT-2 inhibitor, dapagliflozin, on left ventricular remodeling in patients with type 2 diabetes and HFrEF. 60 patients were randomized (1:1) to receive dapagliflozin 10 mg once daily, or placebo double blind for 1 year. Patients underwent transthoracic echocardiography and doppler evaluation prior to dapagliflozin initiation and at 1 year. At 1year, adjusted mean difference versus placebo in change from baseline in LVEF was 2.5% (95% CI: 1.00–4.06, *P* = 0.002). Adjusted mean difference versus placebo in change from baseline in LVED volume was − 6.0ml (95% CI: -8.07 -−3.87, *P*<0.001). Adjusted mean difference versus placebo in change from baseline in LVES volume was − 8.1ml (95% CI: -11.07 -−5.14, *P*<0.001). Similarly, adjusted mean difference versus placebo in change from baseline in LVED diameter was − 1.6 mm (95% CI: -2.67 -−0.62, *P* = 0.002). Adjusted mean difference versus placebo in change from baseline in VTI was 0.20 cm (95% CI: 0.01–0.38, *P* = 0.036). Dapagliflozin was well tolerated. Dapagliflozin was associated with significant and clinically meaningful improvement in echocardiographic parameters versus placebo in patients with type 2 diabetes and HFrEF.

Registration number and date: ChiCTR2300072707, 21/06/2023.

## Introduction

Heart failure with reduced ejection fraction (HFrEF) is a type of heart failure in which the ejection fraction is less than 40%. HFrEF poses a considerable challenge to public health that affects millions of people worldwide. A survey of Chinese heart failure patients found that HFrEF accounted for 39.7% of all heart failure patients [1], indicating that in China, it ranks among the prevalent forms of heart failure.

Research has shown that HFrEF and type 2 diabetes often coexist [2]. In diabetes, cardiac function impairment and cardiomyocyte injury are attributed to the combined effects of various molecular mechanisms. These mechanisms encompass disrupted signal transduction pathways (such as insulin signaling and renin-angiotensin signaling), perturbed metabolism and mitochondrial dysfunction, post-translational modifications of structural and signaling proteins, alterations in cell homeostatic processes like apoptosis and autophagy, endoplasmic reticulum stress, and modifications in gene regulation (including the activation of transcription factors, microRNAs, and epigenetic mechanisms) [3]. On the other hand, the existence of HFrEF can also play a role in the onset and progression of type 2 diabetes mellitus [4]. Heart failure may lead to insulin resistance due to overstimulation of beta-adrenergic receptors [5] and up-regulation of G protein-coupled receptor kinase 2 during ischaemia [6]. This impairs insulin sensitivity, which can set a vicious pathogenetic circle where insulin resistance (IR) exacerbates HF and vice versa. Insulin signalling has protective effects in the heart, including inhibiting apoptosis and oxidative stress [7] and enhancing cardiomyocyte survival during ischaemic injury [8, 9]. Recent findings suggest that improving loading ventricular conditions can restore insulin sensitivity in patients with advanced HF [10]. It is of importance that early detection and aggressive management of both conditions to prevent or delay the onset of complications and improve outcomes for affected individuals.

SGLT-2 inhibitors were initially formulated for the management of type 2 diabetes, constituting a category of medications, but recent studies have shown their potential benefit in HFrEF. Among the SGLT-2 inhibitors investigated in HFrEF, dapagliflozin has been subjected to extensive research, and it has been shown to reduce the risk of heart failure hospitalization and cardiovascular death in patients with HFrEF [11]. Similarly, empagliflozin also reduces the risk of worsening heart failure events in HFrEF patients [12]. What’s even more exciting is that recent researches suggest that dapagliflozin and empagliflozin also provide protection for patients with heart failure with preserved ejection fraction (HFpEF) [13, 14]. This prompts us to further understand the mechanism of action of SGLT-2 inhibitors in treating heart failure. Clearly, this mechanism cannot be solely explained by the glucose-lowering effects of SGLT-2 inhibitors. this prospective study sought to assess the impact of dapagliflozin on echo parameters in patients with type 2 diabetes and HFrEF.

## Study population

In order to be eligible for the study, participants had to meet the following criteria: be at least 18 years old, have a confirmed diagnosis of type 2 diabetes, have an ejection fraction of 40% or lower, and exhibit New York Heart Association (NYHA) class II, III, or IV symptoms. Additionally, Patients were mandated to possess a plasma concentration of N-terminal pro-B-type natriuretic peptide (NT-proBNP) equal to or exceeding 600 pg per milliliter. If patients had atrial fibrillation or atrial flutter upon baseline electrocardiography, their NT-proBNP level needed to be at least 900 pg per milliliter.

### Study design and treatment

Participants who met any of the following criteria were excluded from the study: recent use of or intolerable side effects related to an SGLT2 inhibitor, presence of type 1 diabetes mellitus, hypotension symptoms or a systolic blood pressure below 90 mm Hg, and an estimated glomerular filtration rate (eGFR) lower than 30 ml per minute per 1.73 m^2^ of body surface area (or experiencing rapid decline in renal function).

Patients must have been on stable treatment for at least 3 months prior to recruitment. Then patients were randomized (1:1) to receive dapagliflozin 10 mg once daily, or placebo double blind for 1year; Randomization was undertaken using a computerized permuted-block randomization system (block size of 4) with concealed study group assignments. Throughout the trial, patients maintained their antidiabetes background therapy at a consistent dosage and regimen. Additionally, patients were mandated to undergo standard medication treatment, encompassing an angiotensin-converting-enzyme inhibitor, an angiotensin-receptor blocker, or sacubitril/valsartan in conjunction with a beta-blocker, unless contraindicated or associated with intolerable adverse effects. Furthermore, the utilization of a mineralocorticoid receptor antagonist was recommended. A noteworthy response to dapagliflozin was defined as an (absolute) improvement in LVEF of equal to or greater than 5%.

The primary outcome was the change in LVEF value, and the secondary outcomes were the changes in LVED volume, LVES volume, LVED diameter, and LVES diameter.

The research protocol obtained approval from the ethics committee at Jiangxi Provincial People’s Hospital. The study adhered to the ethical principles specified in the Declaration of Helsinki. Prior to enrollment in the study, participants provided written consent after receiving comprehensive information about the study.

Before initiating dapagliflozin, patients underwent a clinical examination, 12-lead electrocardiography (ECG), transthoracic echocardiography (TTE), and Doppler assessment. The same measurements were repeated after 1 year. The functional evaluation was carried out based on the New York Heart Association (NYHA) classification. TTE was conducted by an observer who was unaware of the patient’s condition. Standard TTE was systematically performed using a commercially available system (Vivid E9, GE Healthcare, France) within 24–72 h prior to dapagliflozin commencement and repeated 1 year after the initiation of dapagliflozin treatment. We have taken similar left echo evaluation in the research conducted by Bayard et al. [15].

### Statistical methods

Before the formal trial commenced, we conducted a preliminary trial involving approximately 8 patients. These 8 patients were randomly assigned to the dapagliflozin treatment group and the placebo group. The preliminary trial lasted for 2 months, and upon its conclusion, an independent samples t-test was performed. The difference in LVEF between the dapagliflozin group and the placebo group was found to be approximately 5%. The standard deviations of LVEF data in both groups were approximately 5%. This preliminary trial provided the basis for selecting the treatment difference and standard deviation for our formal trial.

A sample size of 30 patients per treatment group would provide a power of 90% to detect a treatment difference of 5% in LVEF assuming an SD of 5% at a significance level of 0.05 (two-sided), and a 6.2% dropout rate. Categorical variables in the baseline characteristics are presented as frequencies and percentages, while continuous variables are reported as means ± standard deviation (SD) or medians with interquartile ranges. Differences between groups were evaluated using the independent samples t-test or Mann-Whitney test for continuous variables, and the chi-square test or Fisher’s exact test for categorical variables. Comparison of two groups of echo responders using a chi-square test. Changes in various indices from baseline to 1 year were analyzed using analysis of covariance, with adjusted means (standard error) reported as the results. The analysis of covariance included the baseline value of each analyzed variable as a covariate. Safety analyses were performed on patients who were randomized and received at least one dose of either dapagliflozin or placebo. Statistical analysis was carried out using SPSS 22.0 software. A significance level of P < 0.05 was considered statistically significant.

## Results

**Fig. 1:**
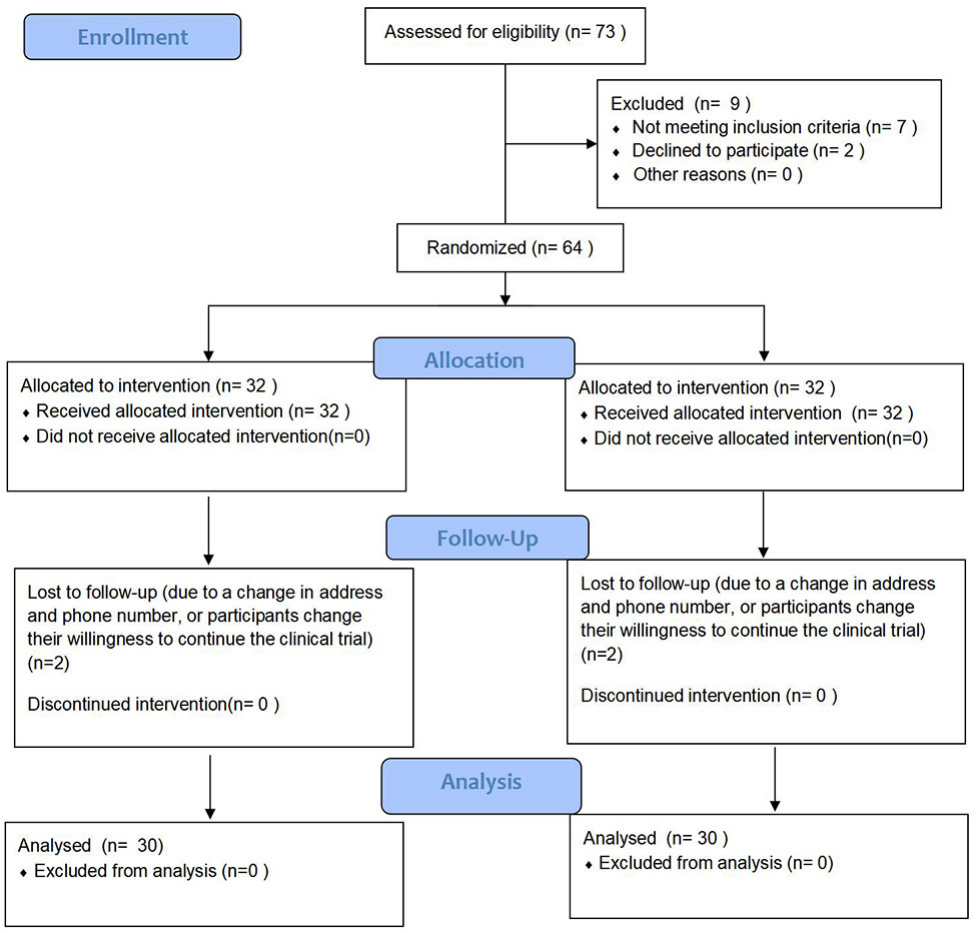
CONSORT flow diagram

Between September 2021 and December 2022, 60 patients with type 2 diabetes and HFrEF were randomized and received study medication. Figure 1 displays the CONSORT flowchart used in the study to depict the recruitment, screening, and follow-up processes of the study. The boxes in the figure represent different steps or phases in the study, while the arrows indicate the flow of information. Readers of the study can use this chart to comprehend the overall flow of the study. Patient baseline characteristics are shown in Table 1. The two patient groups exhibited comparable baseline characteristics. No significant disparities in baseline characteristics were identified between the groups. At 1 year, adjusted mean difference versus placebo in change from baseline in LVEF was 2.5% (95% CI: 1.00–4.06, *P* = 0.002). At 1 year, adjusted mean difference versus placebo in change from baseline in LVED volume was − 6.0ml (95% CI: -8.07 -−3.87, *P*<0.001). Adjusted mean difference versus placebo in change from baseline in LVES volume was − 8.1ml (95% CI: -11.07 -−5.14, *P*<0.001). Similarly, adjusted mean difference versus placebo in change from baseline in LVED diameter was − 1.6 mm (95% CI: -2.67 -−0.62, *P* = 0.002). Adjusted mean difference versus placebo in change from baseline in VTI was 0.20 cm (95% CI: 0.01–0.38, *P* = 0.036). Data are shown in Table 2. After 1 year intervention, echo responders in 15/30 (50%) patients and 10/30 (33.3%) patients in dapagliflozin group and placebo group, respectively (*P* = 0.190).

**Table 1:** Characteristics of the Patients at Baseline

Characteristic	Dapagliflozin (N = 30)	Placebo (N = 30)
Age (yr)	70.7 ± 6.7	70.4 ± 6.3
Female sex— no. (%)	9 (30.0)	8 (26.7)
Body-mass index	24.7 ± 1.5	24.7 ± 1.4
NYHA functional classification (%)		
II	21 (70.0)	20 (66.7)
III	8 (26.7)	8 (26.7)
IV	1 (3.3)	2 (6.7)
Heart rate (beats/min)	75.5 ± 9.8	72.8 ± 10.6
SBP (mm Hg)	114.2 ± 8.3	115.4 ± 9.6
LVEF (%)	30.6 ± 3.8	31.3 ± 3.7
HbA1c (%)	7.9 ± 0.9	8.0 ± 0.8
Median NT-proBNP (IQR) (pg/ml)	1091 (941.8–1643.5)	1088 (1014.5–1516.5)
Principal cause of heart failure (%)		
Ischemic	17 (56.7)	15 (50.0)
Nonischemic	10 (33.3)	11 (36.7)
Unknown	3 (10.0)	4 (13.3)
Atrial fibrillation history	4 (13.3)	3 (10.0)
Estimated GFR (ml/min/1.73 m^2^)	67.5 ± 9.5	67.5 ± 9.6
Heart failure medication (%)		
Loop diuretic	27 (90.0)	28 (93.3)
ACE inhibitor	5 (16.7)	4 (13.3)
ARB	2 (6.7)	3 (10.0)
Sacubitril-valsartan	20 (66.7)	22 (73.3)
Beta-blocker	25 (83.3)	26 (86.7)
Mineralocorticoid receptor antagonist	20 (66.7)	22 (73.3)
Digitalis	4 (13.3)	4 (13.3)
Glucose-lowering medication (%)		
Biguanide	4 (13.3)	5 (16.7)
Sulfonylurea	8 (26.7)	10 (33.3)
DPP−4 inhibitor	6 (20.0)	5 (16.7)
GLP−1 receptor agonist	11 (36.7)	12 (40.0)
Insulin	7 (23.3)	8 (26.7)
LVED volume (ml)	181.6 ± 15.7	178.5 ± 13.5
LVES volume (ml)	126.1 ± 14.5	122.6 ± 11.2
LVED diameter (mm)	59.3 ± 3.9	59.9 ± 6.0
LVES diameter (mm)	50.3 ± 3.8	50.7 ± 4.9
VTI (cm)	16.3 ± 2.4	16.5 ± 2.9
Cardiac index output (L/min/m^2^)	2.6 ± 0.56	2.5 ± 0.44
Systolic pulmonary pressure (mmHg)	31.3 ± 4.7	31.9 ± 5.9
Mitral insufficiency (≥ II Grade) (%)	16 (53.3)	14 (46.7)

LVEF: left ventricular ejection fraction; VTI: velocity time integral; LVED: left ventricular end-diastolic; LVES: left ventricular end-systolic

**Table 2:** Change in characteristics at 1 year

	Dapagliflozin (N = 30)	Placebo (N = 30)
LVEF (%)	36.3 (0.54)	33.7 (0.54)
Change from baseline	5.5 (0.73)	2.5 (0.71)
Difference vs. placebo		2.5 (0.77)
95% CI		1.00–4.06
*P*		0.002
LVED volume (ml)	169.3 (0.74)	175.3 (0.74)
Change from baseline	−12.3 (3.07)	−3.3 (2.30)
Difference vs. placebo		−6.0 (1.05)
95% CI		−8.07 -−3.87
*P*		<0.001
LVES volume (ml)	107.9 (1.04)	116.1(1.04)
Change from baseline	−18.1 (2.66)	−6.7 (2.23)
Difference vs. placebo		−8.1 (1.48)
95% CI		−11.07 -−5.14
*P*		<0.001
LVED diameter (mm)	53.8 (0.83)	55.5 (0.36)
Change from baseline	−5.4 (0.83)	−4.4 (1.08)
Difference vs. placebo		−1.6 (0.51)
95% CI		−2.67 -−0.62
*P*		0.002
LVES diameter (mm)	46.0 (1.04)	48.3 (1.04)
Change from baseline	−4.3 (1.58)	−2.4 (0.97)
Difference vs. placebo		−2.3 (1.47)
95% CI		−5.25 -−0.63
*P*		0.121
VTI (cm)	17.1 (0.07)	16.9 (0.07)
Change from baseline	0.79 (0.45)	0.40 (0.50)
Difference vs. placebo		0.20 (0.09)
95% CI		0.01–0.38
*P*		0.036
Cardiac index output (L/min/m^2^)	2.9 (0.06)	2.7 (0.06)
Change from baseline	0.3 (0.06)	0.2 (0.06)
Difference vs. placebo		0.15 (0.08)
95% CI		−0.02–0.31
*P*		0.076
Systolic pulmonary pressure (mmHg)	23.2 (0.66)	24.8 (0.66)
Change from baseline	−8.0 (1.15)	−7.1 (1.60)
Difference vs. placebo		−1.6 (0.93)
95% CI		−3.44–0.28
*P*		0.094
HbA1c (%)	7.3(0.05)	7.9(0.05)
Change from baseline	−0.6(0.04)	−0.08(0.06)
Difference vs. placebo		−0.6(0.07)
95% CI		−0.702–0.437
*P*		< 0.001

The number of patients with AEs is summarized in Table 3. Events consistent with volume depletion was reported by one patient in dapagliflozin group and in placebo group, respectively (hypotension and orthostatic hypotension). Confirmed hypoglycemic AEs was reported in one patients receiving dapagliflozin. The percentage of patients with events consistent with UTI was reported in more patients receiving dapagliflozin than placebo. The percentage of patients with events consistent with genital infection was higher with dapagliflozin than placebo.

**Table 3:** Summary of AEs

	Dapagliflozin (N = 30)	Placebo (N = 30)
Hypoglycemia	1 (3.3)	0
Events consistent with urinary tract infection		
Male	0	0
Female	2 (6.7)	0
Events consistent with genital infection		
Male	0	0
Female	1 (3.3)	0
Events consistent with volume depletion	1 (3.3)	1 (3.3)

## Discussion

The transportation of glucose and sodium into the proximal tubule cell occurs through the SGLT transport mechanism, where one glucose molecule is coupled with one sodium ion. By inhibiting the SGLT2 protein, sodium reabsorption in the nephron is diminished, resulting in a mild diuretic effect. The presence of elevated glucose in the filtrate leads to osmotic diuresis, which helps maintain increased urine volume [16]. In the DAPA-HF study, SGLT-2 inhibitor, dapagliflozin, reduced the death from cardiovascular causes by 18% and hospitalization for heart failure by 30% in patients with HFrEF [11]. In the EMPEROR-Reduced trial, empagliflozin was also found to have similar protective effects [12]. Therefore, in 2022, the American Heart Association (AHA)/American College of Cardiology (ACC)/Heart Failure Society of America (HFSA) guidelines for heart failure management have included SGLT-2 inhibitors as a standard treatment for patients with HFrEF [17]. Furthermore, in the 2023 ESC Guidelines for the management of cardiovascular disease in patients with diabetes, it is strongly recommended that SGLT-2 inhibitors be the first-choice antihyperglycemic therapy for patients with type 2 diabetes mellitus and atherosclerotic cardiovascular disease (ASCVD) [18].

The alteration of ventricular structure is a significant factor impacting patient morbidity and long-term prognosis. The amelioration of left ventricular remodeling was accompanied by enhancements in left ventricular systolic function. By delaying ventricular remodeling, the progression of heart failure can be fundamentally postponed [19]. This study suggests that dapagliflozin has an additional effect on left ventricular remodeling in patients with HFrEF, even when these patients receive other standardized treatments for heart failure.

In a recent study, it was observed that pigs treated with empagliflozin demonstrated a higher left ventricular ejection fraction and exhibited significantly greater contractile reserve compared to the control animals [20]. Consistent with our research findings, in Otagaki et al.‘s study, the SGLT-2 inhibitor tofogliflozin significantly improved LVEF in patients with type 2 diabetes (5.0 ± 6.9% vs. -0.6 ± 5.5%, P = 0.006) [21]. However, in Cohen et al.‘s study, the mean LVEDV of the treatment group decreased by -10.1ml, while the mean LVEDV of the control group increased by 5.2ml, with a statistically significant difference after 6 months of intervention with empagliflozin. However, there was no difference in LVEF between the two groups [22]. The reason for this difference may be that, in addition to the different examination methods, the enrolled population in the above study were patients with type 2 diabetes. In contrast, the enrolled population in our study were patients with type 2 diabetes complicated by HFrEF. The difference in the results of the two studies seems to indicate that SGLT-2 inhibitors have more meaningful clinical effects in patients with type 2 diabetes complicated by HFrEF. Similarly, in Bonora’s study, there was no statistical difference in cardiac contractile function parameters such as cardiac output, cardiac index, and EF in two groups of type 2 diabetes patients after 12 weeks of dapagliflozin intervention [23]. Again, in the DAPACARD trial [24], Oldgren et al. recruited 53 patients with type 2 diabetes who had normal cardiac function. After administering dapagliflozin intervention for 6 weeks, they did not observe statistically significant improvements in cardiac function among the patients. Besides the relatively short intervention duration, this seems to further reinforce our conclusion that SGLT-2 inhibitors have more meaningful clinical efficacy in improving cardiac remodeling in type 2 diabetes patients with HFrEF, rather than in those with diabetes alone.

In the REFORM trial [25], Singh et al. used cardiac magnetic resonance imaging to assess the impact of dapagliflozin on left ventricular remodeling in patients with type 2 diabetes and heart failure. Unlike the clinical study mentioned earlier, this trial also recruited patients with type 2 diabetes and heart failure, and the intervention duration was one year. However, this trial did not find any benefit of dapagliflozin on left ventricular remodeling in such patients. One possible reason for this is that the trial recruited patients with a baseline LVEF value of approximately 45%, and the majority (87.5%) of patients were classified as NYHA class I or II. In contrast, in our study, patients had a baseline LVEF value of around 30%, and the majority (over 90%) were classified as NYHA class II or III. This suggests that the patients we recruited had worse cardiac function. The difference between the two clinical studies may indicate that the more severe the heart failure in patients, the more dapagliflozin can play a role in improving left ventricular remodeling.

In the clinical trials EMPA-TROPISM [26], SUGAR-DM-HF [27], and Empire HF [28], researchers consistently found that empagliflozin can improve left ventricular remodeling in patients with HFrEF, whether or not they have type 2 diabetes. Despite the use of different SGLT-2 inhibitors in these studies, they still arrived at conclusions consistent with our research findings. It appears that this protective effect is related to the drug class rather than the specific drug variant. This suggests that drugs of this class may share common mechanisms of action or biological effects, enabling them to have similar effects in the treatment of heart failure. This observation is of significant importance for understanding how these drugs work and providing additional treatment options for patients.

Our study is one of the related studies on the effects of SGLT-2 inhibitors on cardiac structure and function in patients with type 2 diabetes and HFrEF. The results support that dapagliflozin can comprehensively improve cardiac remodeling in patients with type 2 diabetes and HFrEF, and has good safety. Daily 10 mg dapagliflozin had no statistically significant difference in adverse events compared to placebo.

Given that study has shown that high-dose SGLT-2 inhibitor can further improve LVEF and LVEDD in HFrEF patients compared to standard dose [29], it is necessary to further explore the optimal dosage of dapagliflozin for patients with type 2 diabetes and HFrEF as well as conduct related safety assessments in future research. Additionally, due to recent studies demonstrating that dapagliflozin and empagliflozin also reduce the risk of worsening heart failure events in patients with HFpEF [13, 14], investigating whether dapagliflozin has similar effects on left ventricular remodeling in HFpEF patients is one of our future research directions.

Another point to mention is that left ventricular mass (LVM) is one of the key parameters for assessing heart disease and it is also one of the important indicators of left ventricular remodeling. Abnormal LVM can predict the risk of cardiovascular events. Furthermore, during the treatment of heart disease, improvements in LVM often indicate the effectiveness of treatment. In EMPA-HEART CardioLink-6 clinical trials [30], patients with type 2 diabetes and coronary artery disease who were treated with empagliflozin for six months, as evaluated by cardiac MRI, showed a clinically significant decrease in LVMi. This may also be one of the mechanisms by which SGLT-2 inhibitors reduce cardiovascular events and heart failure hospitalization rates. However, our study lacked LVM data, which is a limitation of this research. One reason is that echocardiography is less sensitive to changes in LVM compared to cardiac MRI. In future research, we can consider using cardiac MRI methods to assess indices of left ventricular remodeling and include LVM as an observational parameter to make our research data more accurate, comprehensive, and robust.

One final point worth noting, and a relatively significant limitation of our study, is the issue concerning sample size calculation. LVEF change of 3% [31] is the most distinguishing factor for assessing drugs’ positive effects on mortality. This is also the LVEF change value recommended by Grothues for power studies [32]. Furthermore, Kramer et al. confirmed that a 3% change in LVEF is associated with a 20% improvement in mortality [31]. Our study used a 5% change in LVEF as the basis for sample size calculation, which was derived from our preliminary trial results. However, it is essential to emphasize that this statistical approach has certain limitations. Our preliminary trial had a relatively small sample size (8 cases) and a short observation period (2 months), making the results less stable and more susceptible to random factors. Differences in disease severity and other factors may have influenced the outcomes. Moreover, data from small sample sizes are more vulnerable to measurement errors. Therefore, we must acknowledge that using the preliminary trial results as the basis for sample size calculation in our formal study is a limitation of our research. In future studies, it would be advisable to adopt the widely recognized LVEF change value of 3% as the basis for sample size calculation.

## Data Availability

The datasets used and/or analysed during the current study available from the corresponding author on reasonable request.
